# Cortical brain structure and sexual orientation in adult females with bipolar disorder or attention deficit hyperactivity disorder

**DOI:** 10.1002/brb3.998

**Published:** 2018-05-29

**Authors:** Christoph Abé, Qazi Rahman, Niklas Långström, Eleonore Rydén, Martin Ingvar, Mikael Landén

**Affiliations:** ^1^ Department of Clinical Neuroscience Karolinska Institutet Stockholm Sweden; ^2^ Department of Psychology Institute of Psychiatry King’s College London London UK; ^3^ Department of Neuroscience Uppsala University Uppsala Sweden; ^4^ Department of Medical Epidemiology and Biostatistics Karolinska Institutet Stockholm Sweden; ^5^ Institute of Neuroscience and Physiology University of Gothenburg Gothenburg Sweden

**Keywords:** attention deficit disorder with hyperactivity, bipolar disorder, cerebral cortex, health disparities, homosexuality, sexual orientation

## Abstract

**Background:**

Nonheterosexual individuals have higher risk of psychiatric morbidity. Together with growing evidence for sexual orientation‐related brain differences, this raises the concern that sexual orientation may be an important factor to control for in neuroimaging studies of neuropsychiatric disorders.

**Methods:**

We studied sexual orientation in adult psychiatric patients with bipolar disorder (BD) or ADHD in a large clinical cohort (*N* = 154). We compared cortical brain structure in exclusively heterosexual women (HEW,* n* = 29) with that of nonexclusively heterosexual women (nHEW,* n* = 37) using surface‐based reconstruction techniques provided by FreeSurfer.

**Results:**

The prevalence of nonheterosexual sexual orientation was tentatively higher than reported in general population samples. Consistent with previously reported cross‐sex shifted brain patterns among homosexual individuals, nHEW patients showed significantly larger cortical volumes than HEW in medial occipital brain regions.

**Conclusion:**

We found evidence for a sex‐reversed difference in cortical volume among nonheterosexual female patients, which provides insights into the neurobiology of sexual orientation, and may provide the first clues toward a better neurobiological understanding of the association between sexual orientation and mental health. We also suggest that sexual orientation is an important factor to consider in future neuroimaging studies of populations with certain mental health disorders.

## 1 INTRODUCTION

Nonheterosexual individuals have substantially higher risks for common psychiatric morbidity including mood disorders, anxiety disorders, and attention deficit hyperactivity disorder (ADHD; Branstrom, 2017; Frisell, Lichtenstein, Rahman, & Langstrom, 2010; Sandfort, de Graaf, Bijl, & Schnabel, 2001). For example, the risks for depression and anxiety are 1.5 to 2.6 times higher in lesbian, gay, and bisexual (LGB) individuals compared with heterosexual individuals (King et al., 2008). Bisexual individuals, that is, persons with nonexclusive same‐ or opposite‐sex interests or behavior, may be at even higher risk (Semlyen, King, Varney, & Hagger‐Johnson, 2016). Conversely, higher prevalence of same‐sex sexual behavior has been observed in clinical psychiatric patients. For instance, more adults with ADHD identified themselves as bisexual compared with individuals without ADHD (Barkley, Murphy, & Fischer, 2008). As people who identify as LGB individuals make up approximately 3.5% of the population (Gates, 2011, 2014), these mental health disparities bear significantly on the public health burden. In combination with the growing evidence for brain characteristics related to both psychiatric disorders and sexual orientation (reviewed below), this also suggest that sexual orientation may be an important factor to consider in neuropsychiatric research.

Several theoretical mechanisms have been proposed to explain psychiatric health disparities related to sexual orientation. One is *minority stress*, which proposes that social stigma and victimization experienced by nonheterosexual individuals cascades into poorer psychiatric health, involving cognitive processes such as poor emotion regulation and rumination (Branstrom, 2017; Frisell et al., 2010; Meyer, 2003; Sandfort et al., 2001). Another suggested mechanism involves shared underlying factors (e.g., personality traits, genetic traits, hormonal factors) across nonheterosexual sexual orientation and psychiatric ill‐health. For example, ADHD and bipolar disorder (BD) are associated with higher novelty‐seeking traits, which may increase the likelihood of same‐sex sexual experiences (Park, Suh, Lee, & Lee, 2016; Zaninotto et al., 2016). Studies have also suggested shared genetic traits across same‐sex sexual orientation and affective disorders (Zietsch et al., 2012), and neurobiological mechanisms that influence sexual orientation may impact the predisposition to psychiatric morbidity (Sandfort et al., 2001).

Sexual orientation has been associated with structural and functional cerebral characteristics (Abé, Johansson, Allzen, & Savic, 2014; Berglund, Lindstrom, & Savic, 2006; Hu et al., 2008; Kranz & Ishai, 2006; Ponseti et al., 2006, 2007; Rahman, 2005; Savic & Lindström, 2008; Zuloaga, Puts, Jordan, & Breedlove, 2008) believed to result from genetic and/or sex hormone effects similar to those involved in brain sexual dimorphism (Bao & Swaab, 2011; Langstrom, Rahman, Carlstrom, & Lichtenstein, 2010; Rahman, 2005; Sanders et al., 2017). Homosexual men and women appear to show a “cross‐sex shift” of brain structure and function; homosexual men being more female‐typical and homosexual women being more male‐typical (Rahman, 2005; Xu, Norton, & Rahman, 2017). Findings supportive of this include volumetric patterns of brain asymmetry (Savic & Lindström, 2008), gray matter volumes in the perirhinal cortex (Ponseti et al., 2007), and cortical thickness in orbitofrontal and visual areas (Abé et al., 2014). However, it is noteworthy that the literature on this topic is scarce, studies were small, results preliminary, and the field would benefit from replication efforts. Among patients with BD and ADHD, several studies have indicated cortical abnormalities. Adults with ADHD have smaller gray matter volumes primarily in frontal brain regions (De La Fuente, Xia, Branch, & Li, 2013; Frodl & Skokauskas, 2012; Moreno‐Alcazar et al., 2016), although there may also be global cerebral volume reductions (Maier et al., 2015). BD patients generally display smaller cortical volume and thickness in frontal, temporal, and medial occipital regions compared with nonpsychiatric controls (Abé et al., 2015; Hanford, Nazarov, Hall, & Sassi, 2016; Lim et al., 2013; Reavis et al., 2016). Interestingly, Mackay et al. (2010) suggested that the sex difference in brain volume (males greater than females) is accentuated in BD and that the sex difference in lateralisation (females more symmetric than males) is diminished in BD.

Thus, structural brain features of ADHD and BD may partly overlap with those associated with sexual orientation. However, the specific shared and distinguishing features remain unknown and are important to clarify. If nonheterosexual sexual orientation is more prevalent in patients with BD or ADHD, and sexual orientation‐related brain differences exist in clinical patient cohorts, previous neuroimaging studies on BD and ADHD might to some extent have been confounded by differences in sexual orientation.

### 1.1 Aims of the study

In this exploratory study, we aimed to investigate the prevalence of nonheterosexuality in bipolar disorder and ADHD and, for the first time, to quantify sexual orientation‐related cortical differences in these cohorts. To this end, we assessed sexual orientation in our well‐characterized, clinical cohort of individuals diagnosed with bipolar disorder or ADHD, and compared cortical brain structures between heterosexual and nonheterosexual patients. Based on the extant literature, we expected a higher number of nonheterosexual individuals in our patient cohort and that sexual orientation‐related cortical differences (namely, a cross‐sex shift) would be observed in perirhinal, orbitofrontal, and/or medial occipital brain areas.

## 2 METHODS

### 2.1 Participants

Patients were recruited from the St. Göran Project, which provides assessment, treatment, and follow‐up of adult patients with BD or ADHD within the Northern Stockholm Mental Health Service. Persons with BD were recruited during 2005–2016 from the bipolar outpatient unit at the Northern Stockholm psychiatric clinic, Stockholm, Sweden. The baseline investigations have been outlined in detail in previous publications (Abé et al., 2015; Jakobsson et al., 2013). In brief, diagnoses were established using a structured psychiatric interview including the Affective Disorder Evaluation (ADE; Sachs et al., 2003), and the Mini International Neuropsychiatry Interview (M.I.N.I.; Sheehan et al., 1998). The final diagnoses were established by a panel of experienced board‐certified psychiatrists at diagnostic case conferences. Brain imaging data comparing cortical structures of persons diagnosed with bipolar disorder type I (BD‐I) or type II (BD‐II) with healthy controls have previously been published (Abé et al., 2015). Patients with a main diagnosis of ADHD were enrolled from an outpatient unit specialized in assessment and treatment of ADHD and related syndromes. A board‐certified psychiatrist interviewed all case individuals according to a structured intake interview. This included the ADE, which had been modified and complemented with DSM‐IV diagnostic criteria for ADHD.

All participants consented to participate orally and in writing, and were not remunerated for participation. The study was approved by the Regional Research Ethics Committee at Karolinska Institutet, Stockholm, Sweden.

### 2.2 Assessment of sexual orientation

Self‐reported sexual orientation was collected as part of a retroactive home survey in 2016 using a four item Kinsey‐type scale asking about participants’ sexual self‐identification, as well as sexual experience, attractions, and fantasies regarding people of the opposite or same sex. Responses were provided on a 7‐point scale ranging from 0 (exclusively heterosexual self‐identification, sexual experience, attraction or fantasies only involving persons of opposite sex) to 6 (exclusively homosexual self‐identification, sexual experience, attractions or fantasies only involving persons of same sex). Scores on these four items were averaged to yield an overall sexual orientation score ranging from 0 (here referred to as “exclusively heterosexual”) to 6 (“exclusively homosexual”; Kinsey, Pomeroy, & Martin, 1948), in the following referred to as *Kinsey score*. Of 231 patients, 53 ADHD and 101 BD patients provided sexual orientation information (67% response rate). A Kinsey score of 1 has in some previous studies been the upper limit for the inclusion into a “heterosexual” group, which might be suitable when investigating groups at the poles of sexual orientation scale (e.g., hetero‐ versus homosexual individuals). However, this cutoff has recently been criticized, as individuals who do not score 0 on the Kinsey scale have a sexual profile that clearly differentiates them from exclusively heterosexuals (Bailey et al., 2016; Savin‐Williams & Vrangalova, 2013). Therefore, and to facilitate comparisons to other previous studies, we report percentages of nonheterosexual individuals at different Kinsey score thresholds: at >0, ≥1, and >1.

### 2.3 MRI acquisition

Structural MRI scans were acquired at the MR Research Centre, Karolinska University Hospital, Stockholm, Sweden. T1‐weighted images of BD patients were acquired on a 1.5 T Signa Excite MRI medical scanner (General Electric) equipped with an 8‐channel head coil (3D‐SPGR, TR = 21 ms, TE = 6 ms, FOV = 18 cm, flip angle 30°, acquisition matrix 256 × 256 × 128). ADHD patients were scanned on a 3T General Electric scanner (3D‐BRAVO, TR = 6.4 ms, TE = 2.8 ms, FOV = 24 cm, flip angle 12°, acquisition matrix 240 × 240 × 180). Thus, a two‐level categorical factor was used in statistical analyses as covariate of no interest, distinguishing between different diagnoses, which simultaneously controlled for potential scanner effects (e.g., “0” for ADHD and scanner 1 and “1” for BD and scanner 2). Additional axial fluid attenuation inversion recovery T2‐weighted scans were acquired for examination by senior radiologists to screen for neuropathology.

Sexual orientation and structural MRI data were available for 66 females (24 with a main diagnosis of ADHD and 42 with BD) and 40 males (22 with a main diagnosis of ADHD and 18 with BD). Seven ADHD and five BD male patients reported nonexclusive heterosexuality (Kinsey score >0), and only two ADHD and one BD had scores ≥1. This small number of nonheterosexual men does not permit a cortical analysis with respect to sexual orientation. Therefore, we focused on female participants and compared cortical brain structures of 29 females who reported exclusively heterosexual sexual orientation (average Kinsey scores of 0), with those 37 females with any nonexclusively heterosexual sexual orientation (average Kinsey scores >0, up to 4.5). In the following, we refer to these groups as HEW (“heterosexual women”) and nHEW (nonheterosexual women), respectively.

### 2.4 Image processing and statistical analyses

Image processing was carried out using the automated segmentation and surface reconstruction methods implemented in Freesurfer 5.1, yielding vertex‐wise measures for cortical volume, thickness, and surface area (Dale, Fischl, & Sereno, 1999; Fischl et al., 2004). Depending on the nature of the variable, tests for group differences in demographic, descriptive, and clinical variables were performed using *χ* ^2^, independent sample *t*‐, or nonparametric Mann–Whitney *U* tests in SPSS software version 23. Group comparisons in cortical volume, thickness, and surface area were performed vertex‐wise on the whole brain in a general linear model approach using tools provided by Freesurfer (*mri_glm_fit*). In the main analyses, we compared the main effect of group (HEW vs. nHEW) on cortical structure using *age* (continuous) and *main‐diagnosis* (two‐level categorical factor with “1” for BD and “0” for ADHD) as regressors. To investigate larger scale differences and to avoid possible over‐smoothing, we used a surface‐based smoothing with a full width at half‐maximum of 15 mm, as employed in our previous case–control study (Abé et al., 2015). Correction for multiple comparisons was made using a Monte Carlo clusterwise simulation approach (Hagler, Saygin, & Sereno, 2006) correcting for two spaces (hemispheres) with a cluster threshold of *p* = 0.05.

## 3 RESULTS

### 3.1 Kinsey scores

Fifty‐one percent of the subjects did not have an exclusively heterosexual sexual orientation reflected in average Kinsey scores >0; 30% had average Kinsey scores ≥1 (including 1), and 21% reported average Kinsey scores >1 (excluding 1). In males, 34% reported average Kinsey scores >0, 15% ≥1, and 9% >1. Nonheterosexual sexual orientation was more common in women: 61% reported average Kinsey scores >0, 39% ≥1, 30% reported average Kinsey scores >1 (see Supporting Information Figure S1 for score distributions). These numbers are higher than those arrived at in general population surveys (see references in Sections 1 and 4).

### 3.2 Participant characteristics (MRI study)

HEW females had a Kinsey score of 0 by definition, whereas nHEW scored significantly higher (mean 1.5, *SD* = 1, range 0.25–4.5; see Table 1 and Supporting Information Figure S3 for the score distribution). The HEW group was older than nHEW and contained more persons diagnosed with ADHD. We therefore controlled for age and main diagnosis in the statistical analysis. Both groups contained comparable numbers of BD type I and BD type II patients and were equivalent in body mass index, education level, number of depressive and manic episodes, depression rating scores (MADRS), and intracranial volume. BD patients in both groups were also equivalent in mania rating scores (YMRS). HEW and nHEW did not differ with respect to comorbid psychiatric conditions or medication use, with the exception that there were somewhat more BD patients with comorbid ADHD in the nHEW group. We conducted sensitivity analyses testing the robustness of our findings accounting for these and other clinical variables (Supporting Information Table S2).

**Table 1:** Demographic and clinical characteristics of HEW and nHEW patient groups

Characteristic	Heterosexual (HEW)*n* = 29	Nonheterosexual (nHEW)*n* = 37	HEW vs. nHEW
Kinsey score (range)	0.0	1.5 ± 1 (0.25–4.5)	*p *<* *0.001
Age in years (covariate)	45.3 ± 12.4	33.2 ± 10.7	*p *<* *0.001
BMI in kg/m^2^	25 ± 7	25 ± 6	ns
Education level	3 ± 1	3 ± 1	ns
No. of patients with main diagnosis of BD/ADHD (covariate)	23/6	19/18	*p *=* *0.023
BD‐I vs BD‐II subtype	10/13	11/8	ns
ASRS score	31 ± 16	38 ± 14	*p *=* *0.061
MADRS score (median, interquartile range)	6.7 ± 7.5 (4, 9)	5.5 ± 5.9 (4, 9)	ns
YMRS score in BD only (median, interquartile range)	0.9 ± 1.8 (0, 1)	0.8 ± 1.5 (0,0.8)	ns
Intracranial volume in liters	1.43 ± 0.11	1.46 ± 0.16	ns

Group means ± standard deviations (median, interquartile range) or number of participants are listed. BD‐I, Bipolar disorder type I; BD‐II, bipolar disorder type II; BMI, body mass index; ASRS, Adults ADHD self‐report scale; MADRS, Montgomery–Åsberg Depression Rating Scale; ns, not significant; YMRS, Young Mania Rating Scale. We categorized education level as pre‐high school (1, 9 years), high school (2, average 12 years), less than 3 years of university (3, average 2 years), and 3 or more years of university (4, average 4 years). Significance was determined with *χ* ^2^, *t*‐, or nonparametric Mann–Whitney *U* tests, depending on the nature of the variable. Other clinical variables (comorbidities and medication use) are shown in the supplements.

### 3.3 Cortical differences between heterosexual and nonheterosexual female patients

**Figure 1:**
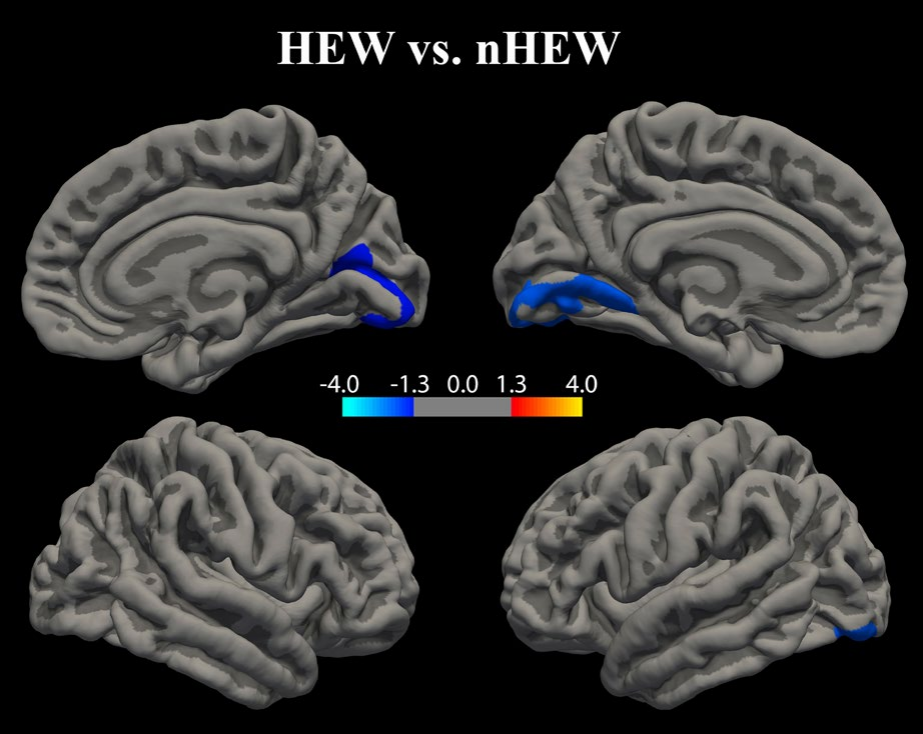
Sexual orientation‐related differences in cortical volume between HEW and nHEW. Clusters with significant cortical volume differences between exclusively heterosexual (HEW,* n* = 29) and nonheterosexual (nHEW,* n* = 37) adult female psychiatric patients. Data underwent Monte Carlo clusterwise correction for multiple comparisons. Significance is displayed on a log(*p*)‐scale where negative values (cold colors) represent the HEW < nHEW cortical volume contrast. No positive values (warm colors) reflecting HEW > nHEW cortical volumes were observed. Clusterwise *p*‐values were *p *=* *0.024 for the cluster in the right hemisphere, and *p *=* *0.003 for the cluster in the left hemisphere. Within each cluster, most significant vertices were found bilaterally in the lingual gyrus (Brodman area 18; see Supporting Information Table S3 in the Online Resource for more details). The extracted mean volume ± standard deviation of the significant clusters was 4,386 ± 705 mm^3^ (HEW) and 5,006 ± 693 mm^3^ (nHEW) in the left hemisphere, with an effect size of 0.89 (Cohen’s *d*). In the right hemisphere, corresponding values were 2,888 ± 561 mm^3^ (HEW) and 3,247 ± 553 mm^3^ (nHEW), with an effect size of 0.64

Significant group effects were observed in left and right medial occipital brain regions. nHEW revealed larger volumes bilaterally in medial occipital cortices compared with HEW. In the right hemisphere, the cluster was mainly comprised of the right pericalcarine sulcus, extending to parts of the cuneus and lingual gyrus. In the left hemisphere, nHEW had larger volumes in a regionally similar cluster, mainly containing the lingual cortex (Figure 1). A similar pattern (nHEW > HEW) within the same brain regions was seen for cortical thickness and surface area, but these differences did not survive correction for multiple comparisons (Supporting Information Figure S2).

### 3.4 Sensitivity analyses and follow‐up testing

To test for potential effects of clinical variables, including comorbid psychiatric conditions and medication use, we performed additional analyses (see Supplemental Material for details). These sensitivity tests supported the robustness of our findings and showed that none of the clinical variables affected the results or the main conclusions.

To test whether nHEW patients show a cross‐sex shift with respect to their cortical structure, we also compared the differing brain areas with those measured in heterosexual male patients (HEM). Overall, these results support a HEW < nHEW <HEM cortical volume pattern, indicating more “male‐typical” structures in nHEW patients (Supporting Information Figure S4).

## 4 DISCUSSION

In this exploratory study, we found that a large proportion of female patients with BD or ADHD (61%) reported a nonheterosexual sexual orientation. We found, for the first time, that nonheterosexual female patients (nHEW) had significantly larger cortical volumes in medial occipital brain regions than exclusively heterosexual female patients (HEW). This suggests that sexual orientation could be a potential confounder in psychiatric neuroimaging research.

### 4.1 Prevalence of sexual orientation

In this study, more than 60% of female and 33% of male patients reported a nonexclusive heterosexual orientation defined as having an average Kinsey score greater than zero. This definition, which captures a broader construct of nonheterosexuality, especially among women, is supported by a large body of research (Bailey et al., 2016): Individuals who score 1 on the Kinsey scale, sometimes called “mostly heterosexual” or having “minor same‐sex attraction,” have a sexual and romantic profile that clearly differentiates them from heterosexuals (Bailey et al., 2016; Savin‐Williams & Vrangalova, 2013). Yet, using a more stringent definition of nonheterosexuality, 30% (9%) of our female (male) patients had an average Kinsey score of >1. According to modern surveys, the Western population prevalence of nonheterosexuality ranges from 1% to 6% when based on identity, 2% to 11% when based on having any same‐sex sexual attraction, and 7% to 9% for any same‐sex sexual interaction (Gates, 2011). Those numbers also are in good agreement with a Swedish survey where only 4% reported strong, and 7% “some” same‐sex attraction (Seto et al., 2010). A study from Ireland that used a Kinsey‐type scale in 2006 found that 94% of women reported “only heterosexual” and 5% “mostly heterosexual” sexual attraction. Only 1.1% scored higher on the scale (Layte, 2006). The most recent study (Britain, 2018) used a five‐point Kinsey‐type scale and reported that 6.5% of males and 11.5% of females reported any same‐sex attraction, which included the points “more often opposite sex” (9.2%), “about equal” (1.1%), “more often same sex” (0.8%), and “same sex only” (0.4%). This study reported comparable numbers with respect to same‐sex experience (Geary & Tanton, 2018). In 2015, surveys from the UK and Israel reported that 16.5%–23% scored greater 0 and 8.5%–14% greater 1 on the Kinsey scale (Panels Research Institute, 2015; YouGov Survey, 2015). However, the sexual orientation questions were based on only one item (self‐identification), and questions about sexual attraction, fantasies, and experience were not assessed, which makes a direct comparison difficult. In addition, the samples in those studies consisted of a significant proportion of young adults, which tended to report minority sexual orientation more often than older adults. Here, we observed a similar tendency as reflected in a negative correlation between Kinsey scores and age (see supporting information). Although somewhat speculative, increasing societal tolerance toward same‐sex sexual orientation might make younger participants provide sexual orientation information more openly. The true reason for this needs to be elucidated. Further, population‐based studies may include individuals with mental health problems, which may further increase previously reported percentages.

Thus, regardless of the threshold used, our results indicate that nonheterosexual sexual orientation, especially in females, may be more prevalent among psychiatric patients than in general population samples. This would be consistent with previous research showing an association between same‐sex orientation and an elevated risk of psychiatric disorders, as well as a previous observation that nonheterosexuality is elevated in ADHD patients (Barkley et al., 2008). However, given the inconsistencies in previously used sexual orientation assessment tools and sample nonhomogeneity, the high prevalence reported here should be treated with caution until further replication.

The reasons for the tentatively high proportion of nonexclusive heterosexual sexual orientation among our study group cannot be clarified in this study, but could be the same factors as proposed in studies addressing poorer psychiatric health in nonheterosexual individuals, for example, minority stress and/or poor social support (Branstrom, 2017; Frisell et al., 2010), shared neurobiological substrates to both same‐sex orientation and mental disorders (Sandfort et al., 2001), and novelty‐seeking behaviors (Park et al., 2016). The latter might, however, only have a limited explanatory effect in our cohort, as sexual experience correlated strongly with the sexual attraction and fantasy subscales of the sexual orientation questionnaire (see Supporting Information Table S2).

### 4.2 Sexual orientation‐related cortical differences

We found significantly larger cortical volumes in nHEW compared with HEW in clusters comprising the calcarine sulcus, and the lingual and cuneus gyri. The effect sizes of the observed differences were medium (right hemisphere) to large (left hemisphere). The differing regions are part of Brodmann areas 17, 18, and 19 that are essential areas of the visual cortex. In fact, sex differences in visual perception and processing have been reported (Handa & McGivern, 2015; Rupp & Wallen, 2008), and several (Abé et al., 2014; Amunts et al., 2007; Raznahan et al., 2010; Ruigrok et al., 2014) but not all (Im et al., 2006; Lv et al., 2010) studies report sex differences in visual brain structures (medial occipital). Amunts et al. (2007) reported sex‐dimorphic features specifically in visual cortical areas (Brodmann areas 17 and 18, containing the lingual gyrus), where males revealed larger volumes than females. This “male greater than female” pattern with respect to cortical volume in medial occipital brain regions has also been observed in a recent meta‐analysis (Ruigrok et al., 2014). In our follow‐up analysis comparing HEW and nHEW to heterosexual male patients (HEM), males revealed greater cortical volumes than both female groups. But interestingly, nHEW had volumes in‐between those of HEW and HEM (Supporting Information Figure S4), suggesting a more “male‐typical” pattern among nHEW. This is also consistent with Abé et al. (2014) who found more “female‐typical” cortical structures in the pericalcarine, cuneus, and lingual cortex among homosexual men. Hence, our results are consistent with the notion of a cross‐sex shift in brain features among nonheterosexual individuals and extend this notion to clinical psychiatric populations and to a broader definition of nonheterosexuality (e.g., including bisexual interests). Moreover, our findings are in line with studies suggesting that sexual orientation influences perception and processing of visual sexual stimuli (Hu et al., 2008; Kranz & Ishai, 2006; Ponseti et al., 2006).

We did not find sexual orientation‐related differences in the perirhinal cortex as reported by one previous study (Ponseti et al., 2007). The perirhinal cortex is, however, connected to the lingual gyrus through the parahippocampal area and also involved in visual perception (Suzuki & Naya, 2014). Moreover, that study compared groups at the poles of sexual orientation (heterosexuals scored 0–1 and homosexuals 5–6 on the Kinsey scale), whereas we used a broader definition of nonheterosexuality. It is possible that exclusive sexual orientation patterns (e.g., homosexuality and heterosexuality) involve different neurobiological pathways than nonexclusive sexual orientation (including bisexuality). Alternatively, sexual orientation could follow a graded, dimensional scale paralleled by neurobiological correlates. Thus, observed differences could be more pronounced in exclusively homosexuals compared with, for example, bisexual individuals. This cannot be elucidated in this study, but requires further research. Depending on brain region, *smaller* volumes found among nonheterosexual women could represent the “male‐typical” pattern (Ponseti et al., 2007; Ruigrok et al., 2014).

The origins of the observed structural brain differences cannot be determined by this study. Previous research on sexual orientation suggests the influence of genetic and environmental factors (Bao & Swaab, 2011; Langstrom et al.; Rahman, 2005; Sanders et al., 2015). Environmental factors proposed to influence sex‐ and sexual orientation‐related neurobiology include pre‐ or postnatal effects of sex hormones (Bao & Swaab, 2011; Goldstein et al., 2001; Hines, 2011; Rahman, 2005). Prenatal exposure to androgens (e.g., testosterone) has not only been linked to sex‐typical behavioral and cognitive traits, but also to masculinization of brain structures, and homosexuality in females (Hines, Constantinescu, & Spencer, 2015; Peplau & Huppin, 2008; Rahman, 2005; Raznahan et al., 2010; Zuloaga et al., 2008). The architecture of the visual cortex is known to be sensitive to environmental influences (Blokland, de Zubicaray, McMahon, & Wright, 2012; Maya‐Vetencourt & Origlia, 2012; Wiesel, 1982), dense in androgen receptors (Goldstein et al., 2001), and might be particularly susceptible to androgen effects in specific periods of development (Bramen et al., 2012; Nguyen et al., 2013; Peper, Hulshoff Pol, Crone, & van Honk, 2011; Raznahan et al., 2010). Indeed, one study found that circulating testosterone levels correlated differently with cortical thickness in calcarine and lingual gyrus in adolescent boys and girls (Bramen et al., 2012). Moreover, sex differences in the visual system have previously been linked to androgen mechanisms influencing visual perception and processing (Handa & McGivern, 2015). Hence, similar effects may play a role in sexual orientation. As sex hormone effects have also been linked to the development of psychiatric disorders (Bao & Swaab, 2011; Martel, Klump, Nigg, Breedlove, & Sisk, 2009; Peper et al., 2011; Sher et al., 2012), one of the overlapping factors influencing both sexual orientation and the vulnerability to psychiatric morbidity could potentially involve sex hormones. Any shared biological mechanisms proposed here may play only a minor role in largely distinct and multifactorial processes (e.g., the interplay of genetic, hormonal, and other environmental factors). It still remains subject to future investigations to identify these factors.

## 5 CONCLUSIONS

This explorative study suggests that rates of nonheterosexuality may be high among females with BD or ADHD. We also detected neurobiological differences between heterosexual and nonheterosexual female patients, where the structural neuroanatomical features of nonheterosexual females were more similar to those of heterosexual male patients. Our study, if replicated in larger samples with matched comparison groups, may contribute to a better neurobiological understanding of sexual orientation and its known association with mental health. The higher prevalence of nonheterosexuality found here also tentatively suggests that future neuropsychiatric studies better control for sexual orientation, especially when including female participants. Conversely, the elevated risk of psychiatric morbidity could impact research addressing sexual orientation etiology. Given potential overlaps, disentangling sexual orientation‐specific from psychiatric neurobiology may improve the accuracy of future neuroimaging studies.

## 6 LIMITATIONS

First, our study was cross‐sectional and does not allow any causal conclusions. Thus, it cannot be determined whether sexual orientation‐related cortical differences are static traits or change during specific developmental stages. Second, although we were able to investigate females, who are underrepresented in research on sexuality, the generalizability to healthy individuals and male patients is limited. Future studies would benefit from using fully factorial designs (e.g., crossing sex, sexual orientation, and psychiatric diagnosis) and longitudinal methods to test for causal pathways. However, those may be difficult to implement in practice, as they require at least eight groups of sufficient sample size. Our cross‐sectional study in a selection of groups of interest (heterosexual female patients, nonheterosexual female patients, and heterosexual male patients) could help clarify the important predictor variables of interest (here sexual orientation) and lend convergent support for hypotheses regarding the pattern of group differences (e.g., cross‐sex shift). Also, cross‐sectional methods are an efficient way to access minority populations with socially stigmatized traits (e.g., sexual orientation and psychiatric health status). Third, we observed a negative correlation between age and self‐reported Kinsey scores (see supporting information). Controlling for age was expected to at least partly control for a potential age‐related reporting bias. Also, when excluding patients older than 50 years the age‐Kinsey score correlation disappeared, and reanalysis with the remaining subsample did not change the results. This and the focality of the observed differences speaks against age as a confounding factor. However, the reason for the observed age‐Kinsey score correlation remains unknown, and the use of self‐report measures has limitations. We cannot exclude the possibility that our data are affected by reporting biases given the sensitive nature of the questions, leading to some underestimation of minority sexual orientation. Thus, it remains unclear how individuals who answered the sexual orientation questionnaire differed from those who did not. The same applies to participants who provided MRI data, and those who did not. Fourth, we combined MRI data obtained from different scanners. This is a standard approach, especially in multicenter studies, as it increases statistical power. We considered the different scanners in the statistical analyses. We also controlled for potential diagnosis effects. The fact that different scanners were used for ADHD and BD patients was a statistical advantage, because the identity of scanner type and diagnosis regressors reduced the number of additional covariates of no interest required from two to one, which increases degrees of freedom and thereby statistical power. It is noteworthy that the same patterns of sexual orientation‐related brain differences were observed in BD and ADHD patients separately (see Supporting Information Figure S5). This, and our statistical control over these factors, speaks against the confounding effects of diagnosis or scanner type. Fifth, we compared the prevalence of nonheterosexuality to the numbers obtained by larger population studies, providing more accurate estimates. However, we want to note that in our case–control study on ADHD‐related neurobiology 8.9% of healthy controls (preliminary *n* = 56, data collection ongoing) reported Kinsey scores greater than 1 (females 11.1% [three of 27], males: 6.9% [two of 29]). This is in good agreement with numbers in aforementioned population‐based studies, including Swedish surveys, supporting our conclusions. However, given the small sample, these numbers should be treated with caution. Similar as in male patients, the small number of nonheterosexuals did not allow any reliable brain structure comparisons.

Finally, although we were able to control for important demographic and clinical variables, it is subject to future studies to test how additional factors such as personality traits (e.g., impulsivity (Ide, Tung, Yang, Tseng, & Li, 2017), social effects (e.g., minority stress factors), or other important biological mechanisms (e.g., genetics and the role of sex hormones) play out.

## CONFLICT OF INTEREST

The authors declare no conflict of interests.
