# Supplementary material for: Cortical brain structure and sexual orientation in adult females with bipolar disorder or attention deficit hyperactivity disorder
Source: Brain Behav. 2018 May 29;8(7):e00998. doi: 10.1002/brb3.998 (PMC6043709; doi:10.1002/brb3.998)
Supplement: Supplementary file 1 [file BRB3-8-e00998-s001.doc]

**Supplementary Material**

**Cortical brain structure and sexual orientation in adult females with bipolar disorder or ADHD**

**Abé et al.**

**Figure S1:** Kinsey score distribution among all male and female patients diagnosed with BD or ADHD who responded to the sexual orientation questionnaire.


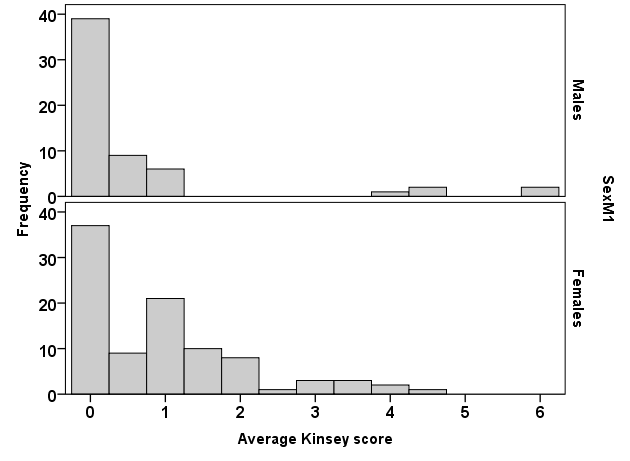


Top: Males (n=59), bottom: females (n=95). In the combined group 50.6% reported Kinsey scores greater than zero reflecting a non-exclusively heterosexual sexual orientation, 29.9% Kinsey scores > 1, and 21.4% reported Kinsey scores >1. In males only, 33.9% reported Kinsey scores >0, 15.3% Kinsey scores >1, and 8.5% Kinsey scores > 1. In females, same sex-attraction was more common: 61.1% reported Kinsey scores >0, 38.9% >1, and 29.5% reported Kinsey scores >1.

**Figure S2:** Differences between HEW and nHEW in *cortical volume* (top)*, thickness* (middle*)* and *surface area* (bottom), uncorrected results (threshold p=0.05).


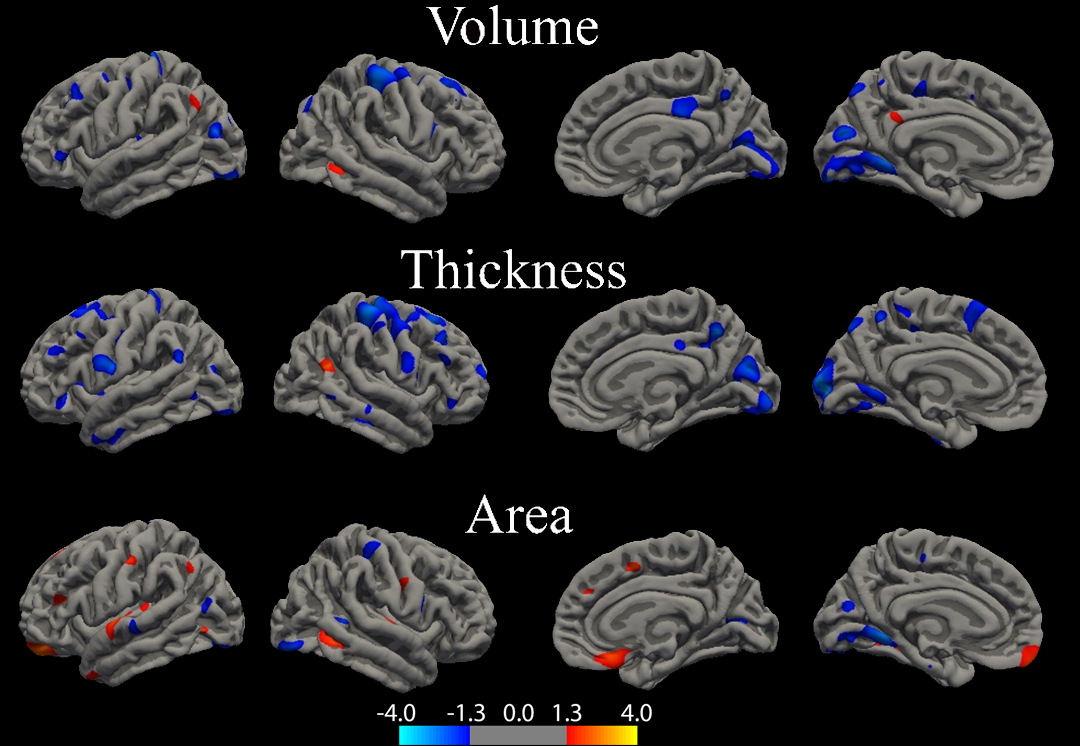


Significance is represented on a log (p-value) scale, where positive values (warm colours) are assigned to HEW>nHEW, and negative values (cold colours) to nHEW>HEW associations. For explorative reasons the display threshold was set to 1.3, corresponding to p < 05 (not corrected for multiple comparisons).

In line with the significant cortical volume differences in medial occipital (visual) areas, thickness and area differences were present in the same regions. Another visually striking cluster was observed in right pre- and postcentral gyrus, where nHEW tend to have larger volume and thicker cortex than HEW.

**Figure S3:** Distribution of Kinsey scores among female patients that provided MRI data, and their correlation with age.


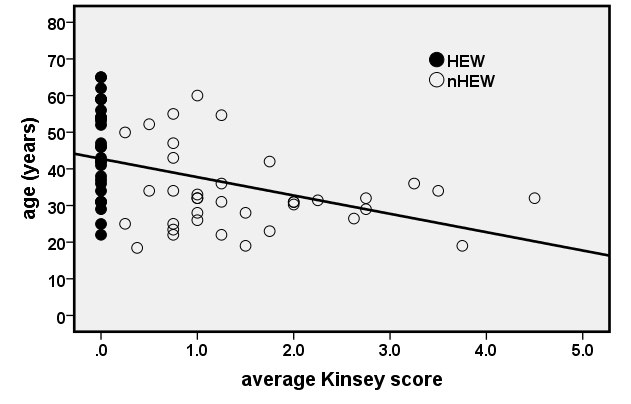


The black dots represent HEW, who had a Kinsey score of 0.0 per definition. White dots represent nHEW. A significant negative correlation between age and Kinsey scores (Spearman’s rho: r=-0.47, p<0.001) was observed.

**Sensitivity analyses and follow-up testing of effects of comorbidity and medication use**

**Table S1: Participants demographics, diagnosis, comorbidities and medication use. Group mean ± standard deviation (median, inter quartile range), and number of participants are listed. Significance was determined with χ^2^, t-tests, or non-parametric Mann–Whitney U-tests, depending on the nature of the variable.**

| **Characteristic** | **HEW (n=29)** | **nHEW (n=37)** | **p** |
| --- | --- | --- | --- |
| age | 45.3 ± 12.4 | 33.2 ± 10.7 | <0.001 |
| Kinsey score (range) | 0 | 1.5 ± 1 (0.25-4.5) | <0.001 |
| No. of patients with BD/ADHD main diagnosis | 23/6 | 19/18 | 0.023 |
| BD-I/BD-II subtype | 10/13 | 11/8 | ns |
| BMI, kg/m^2^ | 25 ± 7 | 25 ± 6 | ns |
| Education level | 3 ± 1 | 3 ± 1 | ns |
| Handedness (right/left/mixed/ missing) | 23/1/0/5 | 25/2/1/9 | ns |
| Intracranial volume in litres | 1.43 ± 0.11 | 1.46 ± 0.16 | ns |
| Years with main psychiatric disorder | 23 ± 14 | 17 ± 10 | 0.076 (ns) |
| No. of depressive episodes (median, interquartile range) | 23 ± 35 (10, 16) | 17 ± 23 (7, 14) | ns |
| No. of manic episodes in BD-I (median, interquartile range) | 3.5 ± 4.6 (1.5,2) | 2.0 ± 1 (2, 2) | ns |
| ASRS score | 31 ± 16 | 38 ± 14 | 0.061 (ns) |
| MADRS score (median, interquartile range) | 6.7 ± 7.5 (4, 9) | 5.5 ± 5.9 (4, 9) | ns |
| YMRS score in BD only (median, interquartile range) | 0.9 ± 1.8 (0, 1) | 0.8 ± 1.5 (0,0.75) | ns |
| No. of current smokers | 8 | 12 | ns |
| No. of current moist snuff users | 2 | 0 | ns |
| Participants with any lifetime history of psychosis | 9 | 10 | ns |
| Medication |  |  |  |
| No. of ADHD patients on central stimulants | 5 | 14 | ns |
| No. of BD patients on lithium | 13 | 13 | ns |
| No. of BD patients on antiepileptic drugs | 13 | 6 | ns (in BD) |
| No. of BD patients on antipsychotic drugs | 3 | 7 | ns |
| No. of patients on antidepressant drugs | 16 | 21 | ns |
| Comorbidity |  |  |  |
| BD with ADHD comorbidity | 3 | 9 | 0.021 |
| ADHD with comorbid mood disorder | 0 | 1 | ns |
| Alcohol use disorder | 4 | 3 | ns |
| Drug use disorder | 0 | 2 | ns |
| Panic disorder | 10 | 11 | ns |
| Social phobia | 3 | 3 | ns |
| OCD | 3 | 2 | ns |
| GAD | 8 | 4 | ns |
| Eating Disorder | 4 | 7 | ns |
| PTSD | 2 | 1 | ns |

We performed sensitivity analyses by repeating the group comparisons on cortical volume extracted from those clusters that showed significant group differences in the main analysis (left and right hemisphere, see Figure 1 in main text). Although most measures were equivalent across groups, the effects of all variables listed in Table S1 on the group comparisons were tested. This was done in separate ANCOVA models using cortical volume as dependent variable, diagnosis and age as covariates, group (nHEW vs. HEW) as fixed factor, and a) the respective test variable in Table S1 as additional covariate, or b) excluding specific cases from the analysis. We separately corrected for body mass index (**BMI), BD subtype (BD-I, BD-II), education, years ill, number of depressive and manic episodes, ASRS, MADRS, YMRS (available in BD only), lithium-, antipsychotic-, antidepressant-use, smoking status, history of psychosis, ADHD comorbidity, panic disorder, generalized anxiety disorder (GAD), and eating disorders. None of these covariates were significant predictors in the statistical model, and neither did adding them as covariate change our results and conclusions. The only predictive variable was intracranial volume (ICV), but using it as a covariate did not change the results (note that groups were equivalent on ICV). In a further series of testing, we excluded patients with specific medication use or comorbidity and repeated the analysis. Hence, our conclusion held when excluding, one at a time, ADHD patients using central stimulants (CS), BD patients using antipsychotics, BD patients with ADHD comorbidity, moist snuff users, participants with alcohol/substance use disorder, social phobia, obsessive compulsive disorder (OCD), GAD, eating disorder, or post-traumatic stress disorder (PTSD). The results also held when excluding left- or mixed handed individuals, or repeating the analysis in individuals of known right-handedness only. These sensitivity tests support the robustness of our findings.**

**Follow-up analyses for interpretational purposes *including heterosexual male (HEM)* patients**

For each participant, cortical measures (volume, average thickness and surface area) were extracted from those clusters that revealed significant sexual orientation-related differences (see Figure 1, main text). To compare the structural similarity to heterosexual men, we compared the outcome measures with those of heterosexual male patients diagnosed with BD or ADHD (HEM). Figure S4 contains the results obtained for these pairwise group comparisons. The male group consisted of 28 patients (13 bipolar disorder, 15 ADHD patients, on average 37 ± 13 years old, Kinsey score = 0). In summary, HEM showed generally larger cortical structures. nHEW had cortical volumes in between that of HEW and HEM. Thickness measures of nHEW were statistically equivalent to those of HEM. nHEW did not differ from HEW in surface area measures. Overall, these results support a HEW<nHEW<HEM pattern, indicating more “male-typical” structures in nHEW.

**Figure S4:** Pairwise group comparisons of *cortical volume (top)* extracted from clusters obtained from the main analysis in left (left panel) and right (right panel) hemispheres. Average *cortical thickness (middle)* and *surface area (bottom)* are shown for completeness. Bars represent means and standard deviations obtained for heterosexual (HEW), non-heterosexual women (nHEW), and heterosexual men (HEM). P-values obtained for pair-wise group comparisons are indicated.


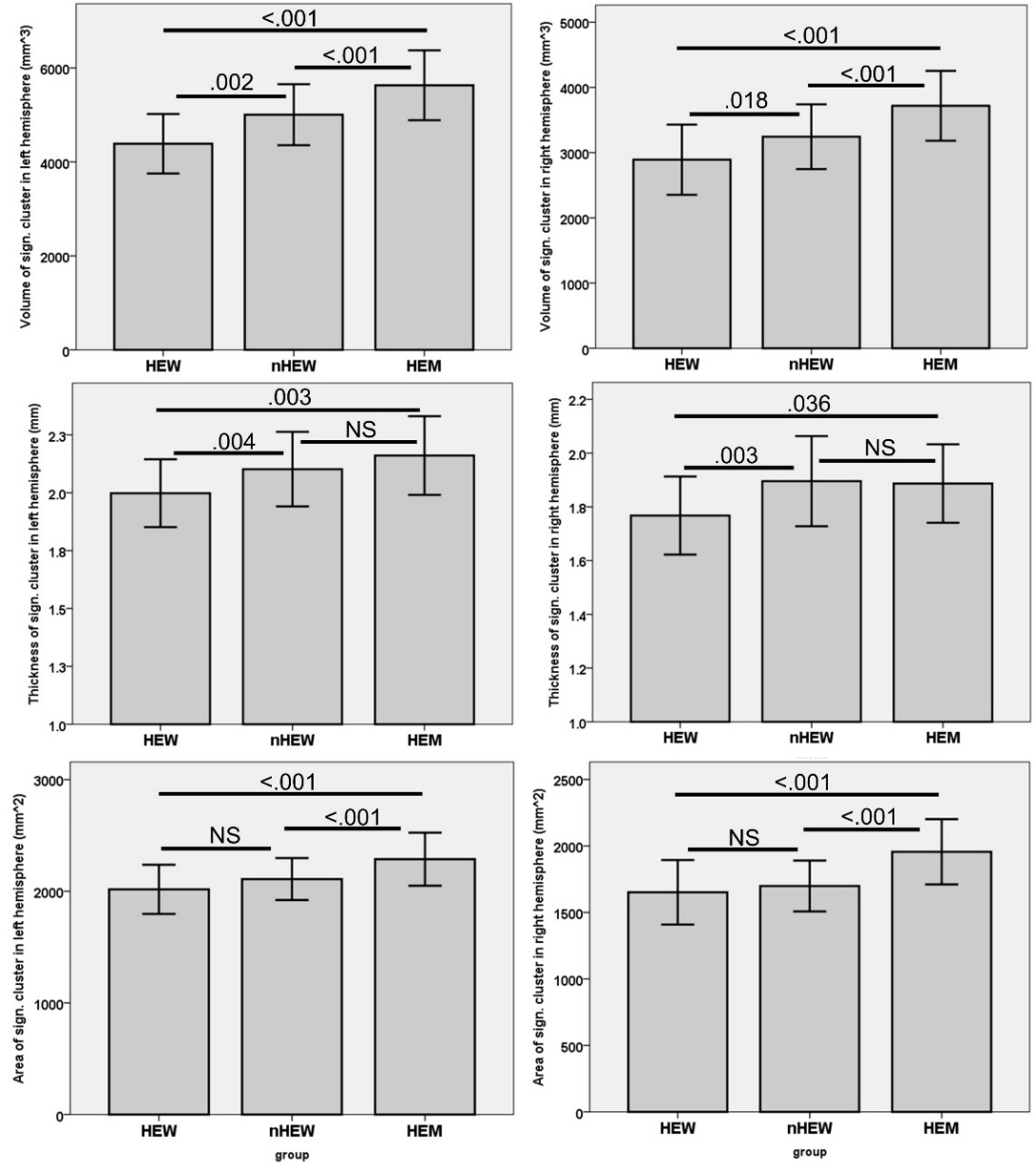


**Figure S5**: Scanner and Diagnosis effects. Group comparisons of *cortical volume* extracted from clusters obtained from the main analysis in left (top panel) and right (bottom panel) hemispheres. Bars represent means and standard deviations obtained for heterosexual (HEW) and non-heterosexual women (nHEW), separated into both patient groups: ADHD scanned at 3T (blue), and BD scanned at 1.5T (green).

With the exemption that the difference in the right hemisphere of BD patients was statistically not significant (most likely because of reduced statistical power), the results and direction of the structural difference obtained within each subgroup were in agreement with the main analysis. Although this significantly reduced the sample size, the tests separately performed within ADHD and BD patients indicate that our findings were unlikely to be influenced by diagnosis or scanner type.


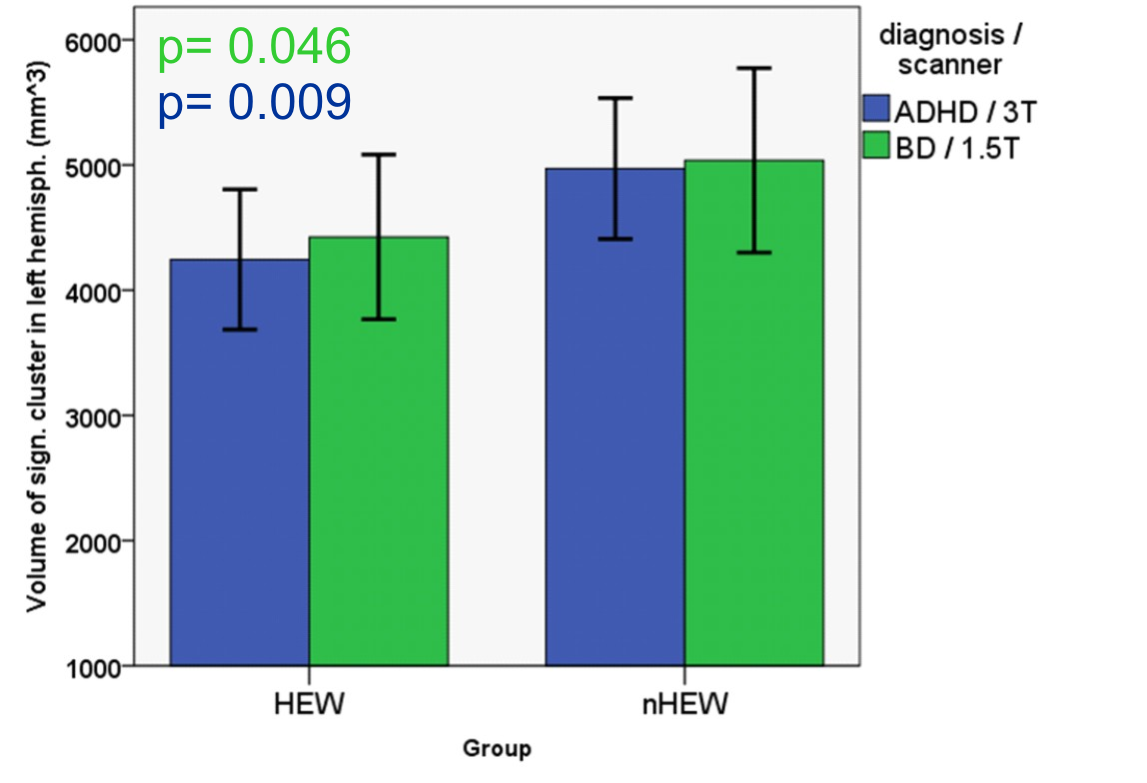


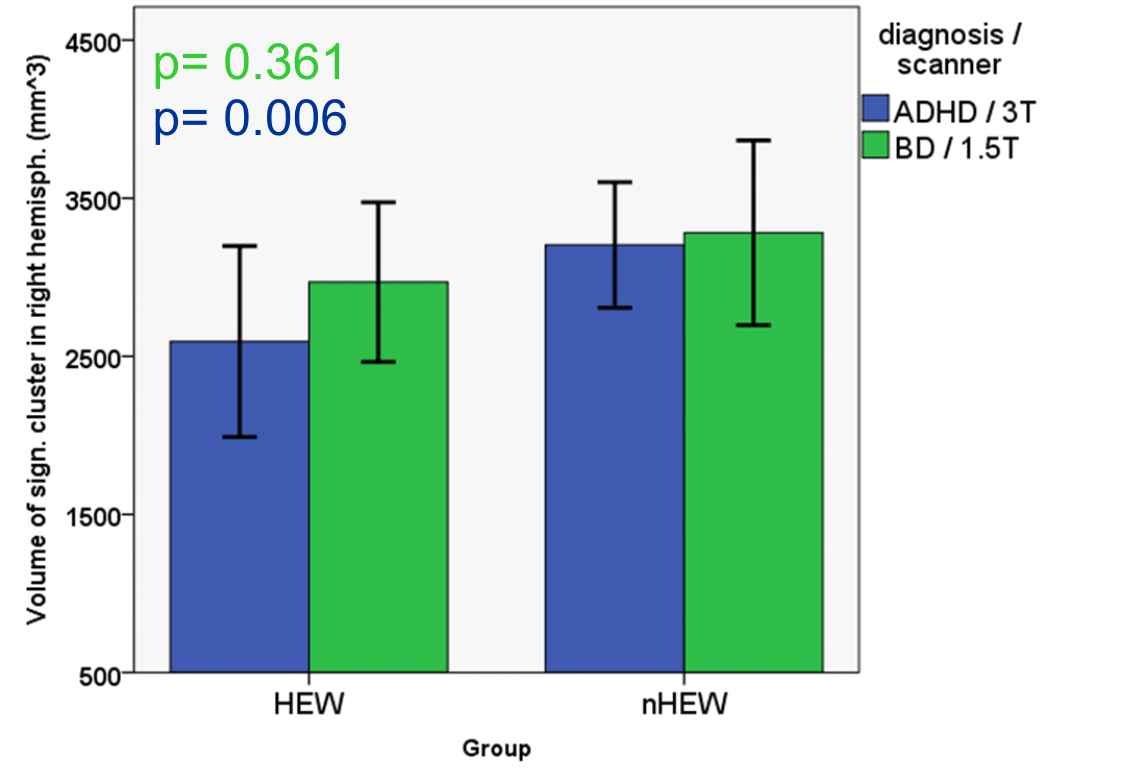


**Table S2:** Inter-correlations (Spearman's rho) between average Kinsey scores and individual items of the sexual orientation questionnaire of n=66 female patients.

|  | **Average score** | **Sexual experience** | **Self-defined sexual orientation** | **Sexual attraction** | **Sexual fantasies** |
| --- | --- | --- | --- | --- | --- |
| **Average score** | 1 | .797** | .790** | .904** | .837** |
| **Sexual experience** | .797** | 1 | .587** | .708** | .501** |
| **Self-defined sexual orientation** | .790** | .587** | 1 | .777** | .556** |
| **Sexual attraction** | .904** | .708** | .777** | 1 | .742** |
| **Sexual fantasies** | .837** | .501** | .556** | .742** | 1 |

**: Correlation significant at p<0.001.

**Table S3: Cluster summary statistics obtained after Monte Carlo cluster-wise correction.**

| Hemisphere | Size(mm^2) | CWP | Max Vertex | Max p | MNI X | MNI Y | MNI Z | AAL label | Brodmann area of cluster peak |
| --- | --- | --- | --- | --- | --- | --- | --- | --- | --- |
| Right | 1557.29 | 0.02425 | -2.372 | 0.004 | 18 | -86 | -11 | lingual | 18 |
| Left | 2062.28 | 0.0028 | -2.712 | 0.002 | -20 | -94 | -12 | lingual | 18 |

**CWP: cluster-wise p-value; Max Vertex: log(p) of most significant vertex within cluster with p-value “Max p” and corresponding MNI coordinates (X, Y, Z). Label obtained from Automated Anatomical Labeling atlas (AAL), and number of Brodmann area is listed.**
